# Dietary Supplement Use during Pregnancy: Perceptions versus Reality

**DOI:** 10.3390/ijerph19074063

**Published:** 2022-03-29

**Authors:** Caihong Xiang, Jing Luo, Guilian Yang, Minghui Sun, Hanmei Liu, Qiping Yang, Yufeng Ouyang, Yue Xi, Cuiting Yong, Muhammad Jamal Khan, Qian Lin

**Affiliations:** 1 Department of Nutrition Science and Food Hygiene, Xiangya School of Public Health, Central South University, 110 Xiangya Rd., Changsha 410078, China; xch0622@csu.edu.cn (C.X.); luojing-csu@foxmail.com (J.L.); minghui.sun@foxmail.com (M.S.); liuhanmeicsu@foxmail.com (H.L.); qipingyang@foxmail.com (Q.Y.); yufeng.ouyang@bristol.ac.uk (Y.O.); xiyue0404@csu.edu.cn (Y.X.); yongcuiting@csu.edu.cn (C.Y.); jamalkhan@csu.edu.cn (M.J.K.); 2 Department of Women Health, Hunan Maternal and Child Health Care Hospital, 53 Xiangchun Road, Changsha 410008, China; yangguiyg@126.com; 3 Centre for Exercise, Nutrition and Health Sciences, School for Policy Studies, University of Bristol, Bristol BS8 1TZ, UK

**Keywords:** dietary supplements, pregnant women, perception, prevalence

## Abstract

This study aimed to examine the prevalence, associated factors and perceptions of dietary supplement use among pregnant Chinese women. A cross-sectional study was conducted to collect data about prevalence, purchase channels, perceptions, and related factors of dietary supplement use from 572 pregnant women, through a face-to-face survey, using a self-designed questionnaire. Of the respondents, 94.8% used at least one dietary supplement, whereas 29.8% used more than four supplements in the previous month. The majority of the pregnant women were highly educated (81.2% had a bachelor’s degree or above) and had the perception that dietary supplements could prevent and improve (89.2%), or treat, nutrition-related diseases (78.7%). Multivariate analysis showed that pregnant women who had used multiple (more than four) supplements were more likely to have a larger gestational age, received fertility treatment, more prenatal visits, and hypothyroidism during pregnancy. Furthermore, pregnant women not only purchased dietary supplements through hospitals (72.6%) and pharmacies (45.1%), but overseas Daigou or online purchases (31.8%) were also a major channel of purchase. A high prevalence of dietary supplement use during pregnancy was observed, with extensive and repeated consumption of nutrients. Pregnant women’s craze for dietary supplements calls for more comprehensive guidelines in China.

## 1. Introduction

Dietary supplements are oral products that contain dietary ingredients, such as vitamins, minerals, amino acids, or other dietary substances, and are used to supplement one’s dietary deficiencies [1]. In the last few decades, dietary supplement use has increased massively worldwide. From 2006 to 2016, China’s global share of dietary supplements rose from 9.6% to 14.3%, second only to the United States (34.0%) [2]. Furthermore, many different types of dietary supplements have been introduced recently and the sales channels for dietary supplements are becoming more and more extensive [3]. People can easily purchase dietary supplements from pharmacies, grocery stores, supermarkets, or online stores, without a doctor’s prescription.

Dietary supplements play an important role in improving maternal health and birth outcomes. Pregnant women have increased nutritional requirements, due to the growth of maternal tissues and fetal development [4], and it can hardly be met through their daily diets [5]. Studies in different countries and regions have found that pregnant women’s intake of micronutrients, such as folic acid, iron and vitamin D, is below the recommended intake [6,7]. Nutritional deficiencies during pregnancy can have serious health consequences, such as iron deficiency, leading to anemia in pregnant women, and folate deficiency, leading to neural tube defects (NTDs) in fetuses [8,9]. In addition to improving the dietary quality of pregnant women, the intake of dietary supplements has become a common approach to increase the intake of certain nutrients.

The use of dietary supplements is quite common among pregnant women worldwide, but the prevalence may vary from country to country. In 2014, A collaborative, multi-center, birth cohort study, conducted in nine European countries, reported the use rate of dietary supplements as high as 97.8% in Spain [10], while in Malawi, Ethiopia, and Tanzania, the use rate of supplements was relatively low, not exceeding 37.0% [11,12,13]. In China, with the improvement of living standards, the popularization of health knowledge and the increased attention to the health of pregnant women and fetuses, the use of dietary supplements during pregnancy has become more and more common [14]. However, few studies have investigated the prevalence of dietary supplement use among pregnant Chinese women, ranging between 28.9% and 93.7% [15,16,17,18], and these studies were not consistent in their definitions of dietary supplements.

Although dietary supplement use has been associated with the improved nutrition and health of pregnant women [19,20], the proper use of dietary supplements during pregnancy is still a concern and has attracted the attention of researchers. Previous studies have shown that certain micronutrients (such as folic acid and iron) consumed by pregnant women have exceeded the upper tolerance limit (UL) [21,22]. Besides this, different studies have only reported the prevalence of dietary supplements used during pregnancy. The reasons for taking dietary supplements, the safety of the purchase channels and whether there is a doctor’s guidance or not are still unknown. The purpose of this study was to explore the prevalence, characteristics, and determinants of dietary supplements used by pregnant women in China. The findings of this study may provide evidence for developing international guidelines related to dietary supplement use during pregnancy and the relevant policies on its sale and promotion.

## 2. Materials and Methods

### 2.1. Sampling Method

A cross-sectional survey was conducted from March to September 2019. Pregnant women who attended the Maternal and Child Health Hospital, Hunan Province, China were included in this survey. According to previous research, the use of dietary supplements among pregnant women in China was about 50%. With an allowable error of 5% and confidence of (1 − α = 0.95), PASS software (PASS 11 citation: Hintze J (2011). PASS 11. NCSS, LLC. Kaysville, UT, USA. http://www.ncss.com/software/pass/procedures/ (accessed on 15 February 2019)) determined that sample size (N) of 402 cases needed to be investigated. Assuming a sample non-response rate of 20%, the sample size or *n* = 402/0.8 = 503 cases. Inclusion criteria: (1) pregnancy; (2) no less than 20 years old; (3) informed consent/voluntary participation; (4) no serious disease or complications; (5) able to read and understand the questionnaire well. Exclusion criteria: low compliance and incomplete questionnaire records (loss of more than 50% of information).

### 2.2. Recruitment

The pregnancy health care clinic of the Maternal and Child Health Hospital, Hunan Province, China was selected as the research site, and pregnant women in the waiting area were the target population. Before the survey, the investigator explained the purpose and significance of the research to respondents, as well as the questionnaire content, time cost, and potential benefits, so that the target population was fully informed. Pregnant women who were willing to participate and sign the informed consent forms were included in this study. During the investigation, participants could withdraw from the study at any time. The study was approved by the Ethics Review Committee of the Xiangya School of Public Health, Central South University (No. XYGW-2019-024).

### 2.3. Data Collection

A self-designed questionnaire was used to collect dietary supplement-related and personal basic information. Trained investigators conducted face-to-face interviews with pregnant women. Information collection was conducted as follows.

#### 2.3.1. Dietary Supplements

We set up two multiple-choice questions to ask participants about their information sources and purchase channels: “What are your sources of information about dietary supplements?” and “Where do you purchase dietary supplements?”. Overseas Daigou is a channel of commerce in which a person outside of China purchases commodities for a customer in mainland China (https://en.wikipedia.org/wiki/Daigou (accessed on 12 February 2019)).

#### 2.3.2. Types of Dietary Supplements Used

Participating pregnant women were asked to recall the frequency of use of various supplements in the past month with response options of “never or less than once/month”, “1–3 times/month”, “1–2 times/week”, “3–4 times/week”, “5–6 times a week”, “1 time/day”, “2 times/day”, and “3 times/day or more”. We investigated the intake of eight categories of dietary supplements among pregnant women, including individual vitamin supplements (folic acid, vitamin D, vitamin C, etc.), individual mineral supplements (calcium, iron, zinc, etc.), and multivitamins, multimineral, multivitamin and mineral supplements (multiple-micronutrient MMN) (containing calcium, iron, zinc, selenium, B vitamins, etc.), herbal medicines (angelica, astragalus, poria, etc.), phytochemicals (grapeseed, resveratrol, lycopene, etc.) and others (Docosahexaenoic acid (DHA), DHA + MMN, amino acids, probiotics, etc.). DHA + MMN were referred to as dietary supplements containing DHA and multiple micronutrients. An additional question was asked to obtain dietary supplements not mentioned above (“Are you taking any dietary supplements not mentioned above?”).

According to the response, the use rate of dietary supplements and repeated use rate of nutrients was calculated. The supplement use rate was defined as the percentage of pregnant women who used dietary supplements ≥1 time in the previous month. The nutrients repeated use rate was defined as the percentage of pregnant women who consumed the same nutrient through more than 2 types of dietary supplements in the previous month. For example, if a pregnant woman used an individual calcium supplement and an MMN supplement within one month, we considered that she had repeatedly supplemented calcium.

#### 2.3.3. Perception of Dietary Supplements

Pregnant women’s perception of dietary supplements was measured using a self-designed questionnaire that contained 9 items. For example, items were “DS can prevent nutrition-related diseases during pregnancy” and “In the case of good nutrition and health during pregnancy, there is no need to DS”. The responses to each question were “strongly disagree”, “disagree”, “neutral”, “agree”, and “fully agree”. The response options, agree and fully agree, were combined as approval.

#### 2.3.4. General Demographic Information and Pregnancy Information

The general demographic data included age, education, household registration, income, employment. We investigated pregnancy information, including pre-pregnancy body mass index (BMI), gestational week, number of fetuses, way of pregnancy (natural pregnancy or with assisted reproductive technology), number of pregnancies, number of deliveries, number of abortions, whether the pregnancy was planned, and antenatal care visits. The data regarding health status during pregnancy were also collected.

Pre-pregnancy BMI was calculated from the pre-pregnancy height and weight, referring to the Chinese BMI classification standard: BMI < 18.5 is underweight, 18.5 ≤ BMI ≤ 23.9 is a healthy weight, 24.0 ≤ BMI ≤ 27.9 is overweight, and BMI ≥ 28.0 is obese [23]. Pregnancy stages were divided into 1–13 weeks as the first trimester, 14–27 weeks as the second trimester, and 28–40 weeks as the third trimester.

### 2.4. Statistical Analysis

EpiData3.1 software was used for data entry and data were analyzed using IBM SPSS (V24.0.). Descriptive data are presented as numbers and percentages. Chi-square analysis of the R × C list was used to compare rates. Binary logistic stepwise regression (Method = Back) was used to analyze the relationship between the demographic characteristics of pregnant women (X) and the number of dietary supplements (Y). Numbers of dietary supplements use ≤3 were assigned 0, and numbers of dietary supplements use ≥4 were assigned 1. A two-tailed *p* < 0.05 was considered statistically in all analyses. There were some missing values in this study (see Table 1, missing data < 10% of total sample size), as participants refused to answer or were unable to recall certain information. Participants who did not provide complete information were excluded from the final analysis.

## 3. Results

### 3.1. Demographic Characteristics

Data regarding demographic characteristics of 572 pregnant women are shown in Table 1. In the first, second, and third trimesters, the proportions of women were 21.5%, 56.5%, and 22.0%, respectively. Among them, 76.6% were 26–35 years old. About 81.2% of respondents held a bachelor’s degree or above, 85.1% were employed, and 52.8% lived in an urban area.

### 3.2. Use of Dietary Supplements

According to the results, 94.8% of pregnant women took at least one type of dietary supplement in the last month, and 29.8% took more than four types. Women with higher educational attainment, higher household incomes, and at their third trimester were more likely to take more supplements (*p* = 0.005, 0.014, 0.001, respectively). Dietary supplement use was positively associated with assisted reproductive technology (ART) use, number of antenatal visits, number of abortions, iron-deficiency anemia, and hypothyroidism (*p* = 0.005, 0.005, 0.010, 0.002, respectively) (Table 1).

**Figure 1:**
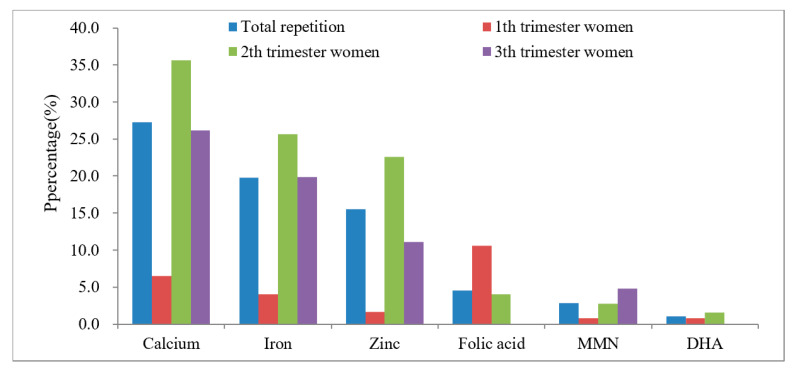
Usage rate of several common dietary supplements in different stages of pregnancy (*n* = 572).

The use of dietary supplements in different stages of pregnancy is shown in Figure 1. The most popular dietary supplement was calcium (65.7%), followed by iron (46.9%), zinc (40.9%), MMN (36.9%), DHA (25.7%), folic acid (21.3%), and MMN + DHA (8.0%). Calcium, iron, and zinc supplements were more prevalent among women at the second and third trimesters, while first-trimester women reported a higher use of folic acid and MMN supplements.

### 3.3. Repeated Supplementation of Nutrients

**Figure 2:**
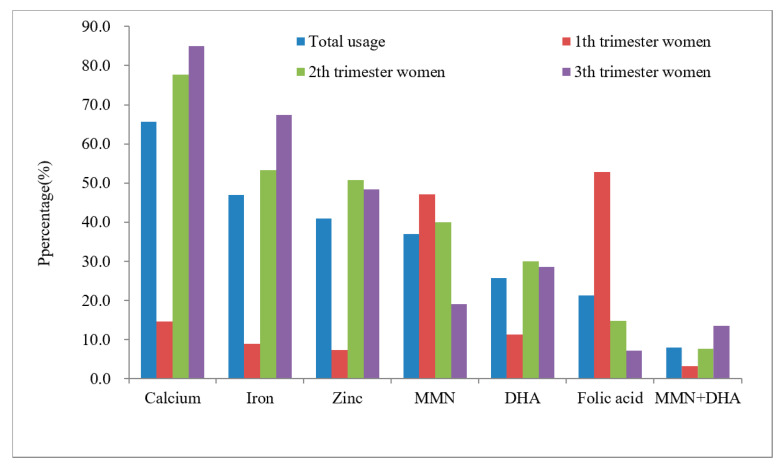
Repeated supplement rate of several common nutrients among pregnant women (*n* = 572).

The repeated supplementation of nutrients among pregnant women is shown in Figure 2. The highest rate of repeated supplementation was for calcium, at 27.3%, followed by iron, zinc, folic acid, MMN, and DHA; the percentages were 19.8%, 15.6%, 4.5%, 2.8%, 1.0%, respectively.

### 3.4. Information Sources and Purchase Channels

Information sources and purchase channels of dietary supplements are provided in Table 2. The sources of information for pregnant women regarding dietary supplements were mainly doctors, family and friends, and Internet knowledge, accounting for 69.9%, 50.0%, and 47.6%, respectively. The data regarding DS purchase revealed that women obtained dietary supplements mainly through hospitals, pharmacies, or overseas Daigou or online purchases, accounting for 72.6%, 45.1%, and 31.8%, respectively.

### 3.5. Pregnant Women’s Perceptions of Dietary Supplement Use

Pregnant women’s perceptions of dietary supplement use are presented in Table 3. Most pregnant women held a positive attitude towards dietary supplement use. The majority of the pregnant women had the perception that dietary supplements could prevent and improve (89.2%), or treat, nutrition-related diseases (78.7%). According to 84.3% of pregnant women, supplements were important. Around 94.1% and 92.3% of respondents, respectively, considered it important to have a basic understanding/knowledge of supplements and consult a professional dietitian or obstetrician before taking them. Only 53.3% of women thought they could judge from the nutrition label of the supplements whether it is suitable for use or not.

### 3.6. Factors Associated with the Use of Multiple Dietary Supplements

Table 4 shows the multivariable analysis of factors associated with dietary supplement use. Comparison based on educational level showed that pregnant women with a bachelor’s degree or above were more likely to use four or more dietary supplements than respondents with lower educational levels (OR = 6.680, 95% CI: 1.776–25.124, *p* = 0.005). Comparison based on the current pregnancy trimester indicated that pregnant women in the second and third trimester were more likely to use four or more dietary supplements (OR = 8.633, 95% CI: 3.690–20.195, *p* < 0.001; OR = 7.293, 95% CI: 2.764–19.248, *p* < 0.001, respectively). Comparison based on the mode of conception revealed that pregnant women who conceived through ART were more likely to use four or more dietary supplements (OR = 3.588, 95% CI: 1.578–8.158, *p* = 0.002). Furthermore, comparison based on antenatal care visits during pregnancy showed that, compared to least visits (≤5 visits to the hospital) more antenatal care visits (6–10 and ≥11 visits) were undertaken by pregnant women who used four or more dietary supplements (OR = 1.665, 95% CI: 1.038–2.671, *p* = 0.035; OR = 2.728, 95% CI: 1.216–6.124, *p* = 0.015, respectively). Moreover, hypothyroidism during pregnancy was more common in pregnant women who used four or more dietary supplements (OR = 1.931, 95% CI: 1.082–3.446, *p* = 0.026).

## 4. Discussion

The key findings of this study were that a high proportion of pregnant women (94.8%) used dietary supplements during pregnancy and around 29.8% of the respondents used four different types of dietary supplements simultaneously. The most popular dietary supplements were calcium (65.7%), followed by iron (46.9%), zinc (40.9%), MMN (36.9%), DHA (25.7%), folic acid (21.3%), and MMN + DHA (8.0%). However, the intake of folic acid in the middle and late stages of pregnancy was considerably low and most pregnant women were not aware of the long-term benefits of folic acid supplementation and its consequences on fetus health in case of deficiency. Beside this, the majority of the pregnant women had the perception that dietary supplements could prevent and improve (89.2%), or treat, nutrition-related diseases (78.7%). Multivariate analysis showed that pregnant women who had used multiple (more than four) supplements were more likely to have a larger gestational age (second and third trimester), received fertility treatment (ART conception), more prenatal visits (6–10 or ≥10 visits) and more chances of hypothyroidism during pregnancy. Furthermore, it was noted that dietary supplements were not only purchased through hospitals (72.6%) and pharmacies (45.1%), but overseas Daigou or online stores (31.8%) were also an important channel of purchase, with no proper security or checks.

Our finding regarding the prevalence of respondents using dietary supplements is significantly higher than reported in a previous study (66.4%), conducted in China in 2009 [24], but is close to the results from another recent study (2019), conducted on 7931 first-trimester pregnant women (93.7%) in China, which is almost similar to the prevalence in several Western countries [10,17,25]. Moreover, it is a common finding that most pregnant women are not limited to only one type of supplement [17,26]. This was the case in this study, where nearly 30% of pregnant women used more than four dietary supplements simultaneously. Due to this, there may be duplication of nutrients in different supplements, increasing the risk of overconsumption [18]. Furthermore, several studies also reported that combining the intake of iron, calcium and magnesium supplementation can produce undesirable interactions, leading to many complications during pregnancy [27,28,29]. That is why it is crucial to monitor supplement consumption among pregnant women, to avoid the potential risks of excessive intake.

According to the results, the most common dietary supplements used by pregnant women were calcium, iron and zinc, especially in the second and third trimesters. During pregnancy, maternal blood volume increases, and serum levels of trace elements, such as calcium, iron, and zinc, decrease, causing deficiency [30,31]. In China, serum calcium, iron, and zinc deficiencies among pregnant women are quite common and adverse effects of these deficiencies on maternal and fetal health have been reported in the past [30,32]. On the other hand, many studies have reported that corresponding nutrient supplementation during pregnancy improves serum levels of iron, calcium and zinc, reducing the chances of deficiency [20,33]. The World Health Organization (WHO) also recommends that pregnant women should ingest 30–60 mg of iron daily, to reduce the risk of low birth weight, maternal anemia and iron deficiency [34], and 1.5–2.0 g of calcium daily, to reduce the risk of pre-eclampsia [35]. However, in China, there is currently a lack of guidelines for micronutrient supplementation, and pregnant women are recommended to fulfill iron and calcium needs from daily food intake [4]. Thus, micronutrient supplementation varies in different geographical regions and socioeconomic groups of the country, and improper DS behavior occurs [36]. As in our study, 15–27% of pregnant women took repeated supplements containing calcium, iron, and zinc and there might be a risk of exceeding the WHO’s recommendations.

Observing the usage of individual DHA or DHA-containing supplements, which was about 33%, this much higher than the 17% reported for Chinese women in 2019, but similar to what was reported in the United States (24%) and Germany (32%) in the TEDDY prospective cohort study and lower than Iceland (50%) and Norway (59%) [10,17,25,37]. Unfortunately, there is still a lack of advice and guidance on the use of DHA supplements during pregnancy in China, and the health benefits of DHA for mothers and infants remain uncertain. The results of systematic reviews in 2016 and 2018 showed that prenatal supplementation of omega-3 long-chain polyunsaturated fatty acids reduced the risk of preterm infants, low-birth-weight infants, and perinatal death, but it also increased the risk of larger gestational age, and reported no effect on the development of vision and cognition after birth [38,39]. Therefore, it is still important to regulate the use of DHA supplements among pregnant women in China, to avoid potential health risks caused by improper use of DHA supplements.

Folic acid is the most commonly recommended dietary supplement during pregnancy, as sufficient evidence has shown that peri-conceptual folic acid intake reduces the risk of neural tube defects [40]. Natural folate intake from daily food with low bioavailability can hardly meet the nutritional requirements of pregnant women. Many countries have issued guidelines for pregnant women, recommending perinatal folic acid supplementation, including China [10,41]. In our results, not surprisingly, the rate of folic acid or MMN containing folic acid supplementation in pregnant women was highest in the first trimester but drastically declined in the middle and last trimester. The same patterns of folic acid supplementation decline were observed in studies conducted in northwestern China and some other countries [4,36,42]. In addition to NTDS, studies found that taking folic acid throughout pregnancy benefited pregnant women and babies in other ways, such as reduction in recurrent pre-eclampsia and better child cognitive development [43,44,45]. As recommended by the Chinese Dietary Guidelines, folic acid supplementation should continue throughout pregnancy [4]. However, our results were in contradiction to these recommendations, as the current use of folic acid supplementation in the middle and late stages of pregnancy was undesirably low. Most pregnant women were not even aware of the long-term benefits. Another concern was that more than 10% of pregnant women in the first trimester of pregnancy retook folic acid from individual micronutrient (IMN) and MMN, which might lead to folic toxicity, increasing the risk of colorectal neoplasia [46,47].

Few studies have investigated the factors associated with multiple dietary supplement use among pregnant women, while most of the studies focused on different characteristics between users and non-users [25,27,42,48,49,50]. In our study, pregnant women who had used multiple supplements were more likely to have higher education, lager gestational age, received fertility treatment, had more prenatal visits and suffered some diseases during pregnancy. These factors are consistent with previous studies that promoted dietary supplement use in pregnant women [25,27,42,48,49,50]. One more interesting association between high education level and multiple supplement use was observed. Education is considered to have an important role in spreading awareness to improve pregnancy-related outcomes. Pregnant women with higher educational attainment are considered to be more aware of the role of nutrition in improving health [25,51], but a lack of accurate guidance might encourage them to use multiple dietary supplements simultaneously, which, in turn, may lead to frequent nutrient overdose, causing problems to the body. However, a recent study (2020) conducted in Queensland, New Zealand, reported no significant relation between educational attainment and the use of both MMN and IMN simultaneously, after adjusting for income and age [26]. Our findings regarding the relation between educational level and multiple dietary supplement use need to be confirmed by more follow-up studies.

During routine prenatal visits and medical consultations, doctors may give respondents information about dietary supplements and their benefits, which may motivate pregnant women to use multiple dietary supplements simultaneously, increasing the chances of unfavorable reactions [52]. Perception investigation of pregnant women suggested high levels of enthusiasm for dietary supplement use, but poor ability to read the nutrition labels. The majority of the respondents believed that dietary supplements could prevent and improve, or treat, nutrition-related diseases that might occur during pregnancy. Even with satisfied nutrition status, they still feel the need to take supplements. This over-confidence might have led them to take multiple dietary supplements simultaneously, without knowing the consequences. Although most women received professional advice from doctors, they still had problems with DS perception and behavior. One reason may be that, in clinical practice, doctors ignored the importance of a detailed assessment of dietary and nutritional status before giving DS recommendations [53]. Another is that the accurate information about DS, such as optimal timing and side effects, has not been effectively disseminated [54]. Still, 30% of the respondents got their opinion from unreliable sources, such as friends and family, newspapers, and the Internet. The massive spread of misinformation, such as exaggerations of treatment effectiveness, can easily mislead consumers into improper DS behavior [55]. An observational study conducted in Chengdu, China, found that more than 50% of pregnant women reported family members as the main source of information [56]. Our study suggested that family based health education may promote the proper use of dietary supplements by pregnant women.

Purchase channels were not limited to hospitals and physical pharmacies only, but also Daigou and online shopping sources, which are quite popular in China, were used for dietary supplementation purchases too [2,3]. Security risks should be considered when purchasing supplements from these sources, as many pregnant women were unable to read product labels and the quality of products was not guaranteed [3,57]. Moreover, countries and regions face different nutritional problems [58,59] and nutritional components contained in overseas products may not be suitable for the nutritional needs of local pregnant women in China. For example, China has implemented the intervention policy of iodized salt, and some pregnant women are at risk of excessive iodine intake [58]. This risk may be exacerbated with dietary supplements produced elsewhere.

This study had certain limitations. Firstly, only a single center was selected for data collection, based on the researcher’s personal contacts and feasibility, which might have increased the chances of selection bias in the study. The intake reported by the study population cannot reflect the intake of dietary supplements among all pregnant women in China, especially among rural women in poor areas. Secondly, although this study investigated the frequency of use of dietary supplements, it did not accurately calculate the intake dose of various nutrients and could not determine whether the intake is appropriate as per guidelines or it exceeds the limit. The data collection of dietary supplement intake was based on respondents’ recollections, rather than daily records, resulting in the possible presence of recall bias. Despite these limitations, our study provided a comprehensive understanding about multiple dietary supplement use and repeated supplementation of the same nutrient among pregnant women, which is a relatively new health-related behavior problem, rarely addressed in previous studies. We assessed perception and purchase behavior, as well as prevalence of dietary supplement intake among pregnant women, which may provide clues for future health interventions related to dietary supplement use.

## 5. Conclusions

The use of dietary supplements during pregnancy is quite high among Chinese women. Most of the respondents believed that dietary supplements could prevent or improve nutrition-related diseases during pregnancy. However, some pregnant women have the problem of taking multiple dietary supplements, with nutrients being repeatedly supplemented. Recommended standards and safety limits regarding dietary supplement intake during pregnancy should be established to improve the nutritional status of pregnant women and reduce potential health risks. Furthermore, policies should be developed to ensure the controlled sale of these dietary supplements.

## Figures and Tables

**Table 1:** Prevalence of dietary supplement use among pregnant women by demographic characteristics (*n* = 572).

		Types of Dietary Supplements Used (*n*,%)				
Characteristics	*n (*%)	0	1–3	4–6	*χ* ^2^	*p*
Total	572 (100%)	30 (5.2)	372 (65.0)	170 (29.8)		
Age (years) *					7.796	0.091
≤25	74 (13.0)	8 (10.8)	51 (68.9)	15 (20.3)		
26–35	437 (76.6)	20 (4.6)	284 (65.0)	133 (30.4)		
36–45	59 (10.4)	2 (3.4)	36 (61.0)	21 (35.6)		
Educational level *					14.072	0.005
Junior high school and below	33 (5.8)	4 (12.1)	25 (75.8)	4 (12.1)		
High school	74 (13.0)	7 (9.5)	51 (68.9)	16 (21.6)		
Bachelor degree and above	461 (81.2)	19 (4.2)	292 (63.3)	150 (32.5)		
Employed during pregnancy *					2.256	0.314
Yes	484 (85.1)	23 (4.8)	314 (64.9)	147 (30.3)		
No	85 (14.9)	7 (8.2)	56 (65.9)	22 (25.9)		
Household register *					1.328	0.515
Rural area	269 (47.2)	17 (6.3)	175 (65.1)	77 (28.6)		
Urban	301 (52.8)	13 (4.3)	195 (64.8)	93 (30.9)		
Income (RMB/month) *					12.480	0.014
2000–5999	97 (17.4)	8 (8.2)	71 (73.2)	18 (18.6)		
6000–9999	213 (38.2)	15 (7.0)	128 (60.1)	70 (32.9)		
≥10,000	247 (44.4)	7 (2.8)	161 (65.2)	79 (32.0)		
Pre-pregnancy BMI *					3.311	0.507
Underweight	97 (17.1)	3 (3.1)	59 (60.8)	35 (36.1)		
Healthy weight	402 (70.9)	23 (5.7)	267 (66.4)	112 (27.9)		
Overweight/Obese	68 (12.0)	4 (5.9)	43 (63.2)	21 (30.9)		
Gestational stage					48.037	<0.001
First trimester	123 (21.5)	4 (3.3)	112 (91.1)	7 (5.6)		
Second trimester	323 (56.5)	19 (5.9)	188 (58.2)	116 (35.9)		
Third trimester	126 (22.0)	7 (5.6)	72 (57.1)	47 (37.3)		
Number of fetuses					3.685	0.148
1	543 (94.9)	30 (5.5)	356 (65.6)	157 (28.9)		
≥2	29 (5.1)	0 (0.0)	16 (55.2)	13 (44.8)		
Way of pregnancy *					9.887	0.005
Natural	503 (90.6)	29 (5.8)	332 (66.0)	142 (28.2)		
ART	52 (9.4)	1 (1.9)	25 (48.1)	26 (50.0)		
Number of pregnancies *					17.673	0.001
1	273 (48.7)	5 (1.8)	189 (69.2)	79 (28.9)		
2	150 (26.7)	15 (10.0)	96 (64.0)	39 (26.0)		
≥3	138 (24.6)	10 (7.2)	79 (57.3)	49 (35.5)		
Number of deliveries *					8.649	0.072
0	351 (62.0)	13 (3.7)	225 (64.1)	113 (32.2)		
1	194 (34.3)	14 (7.2)	129 (66.5)	51 (26.3)		
≥2	21 (3.7)	3 (14.3)	14 (66.7)	4 (19.0)		
Number of abortions *					14.551	0.005
0	378 (67.1)	15 (4.0)	261 (69.0)	102 (27.0)		
1	122 (21.7)	13 (10.7)	71 (58.2)	38 (31.1)		
≥2	63 (11.2)	2 (3.2)	34 (54.0)	27 (42.8)		
Number of antenatal visits *					33.833	<0.001
≤5	284 (54.0)	18 (6.3)	211 (74.3)	55 (19.4)		
6 to 10	192 (36.5)	4 (2.1)	116 (60.4)	72 (37.5)		
≥10	50 (9.5)	3 (6.0)	22 (44.0)	25 (50.0)		
Planned pregnancy *					0.826	0.662
Yes	373 (65.8)	17 (4.6)	243 (65.1)	113 (30.3)		
No	194 (34.2)	12 (6.2)	127 (65.5)	55 (28.3)		
Iron deficiency anemia *					8.948	0.010
Yes	84 (14.7)	0 (0.0)	52 (61.9)	32 (38.1)		
No	487 (85.3)	30 (6.2)	320 (65.7)	137 (28.1)		
Hypothyroidism *					12.368	0.002
Yes	72 (12.6)	1 (1.4)	37 (51.4)	34 (47.2)		
No	499 (87.4)	29 (5.8)	335 (67.1)	135 (27.1)		

ART: Assisted reproductive technology. * There are missing data.

**Table 2:** Information sources and purchase channels of dietary supplements.

Variable		*n*	(%)
Information sources for DS			
	Doctor	400	69.9
	Family or friends	286	50.0
	Network knowledge	272	47.6
	Newspaper or magazine	118	20.6
	Advertising	43	7.6
Purchase channels			
	Hospitals	415	72.6
	Pharmacies	258	45.1
	Overseas Daigou or online purchases	182	31.8
	Community	29	5.1
	Other places	22	3.8

DS: dietary supplement.

**Table 3:** Pregnant women’s perceptions of dietary supplement use.

Item	Approval (%)
DS can prevent nutrition-related diseases during pregnancy.	89.2
DS can improve/treat nutrition-related diseases during pregnancy.	78.7
In the case of good nutrition and health during pregnancy, there is no need to DS.	34.8
In the case of malnutrition symptoms/disease during pregnancy, it is necessary to use DS.	96.0
Before taking DS, it is important to have a basic understanding/knowledge of DS.	94.1
Before taking DS, it is important to consult a professional nutritionist or obstetrician.	92.3
DS can cause nausea, vomiting, and symptoms of constipation and indigestion.	36.7
Taking DS during pregnancy is very important.	84.3
I can judge from the nutrition label of the DS whether it is suitable for my own use.	53.3

DS: dietary supplement.

**Table 4:** Logistic regression analysis results of factors related to pregnant women using multiple dietary supplements *.

Variables	β	Wald χ^2^	OR (95% CI)	*p*
Educational level (vs. Junior high school and below)		11.338		
High school	1.090	2.362	2.975 (0.741, 11.947)	0.124
Bachelor degree and above	1.899	7.894	6.680 (1.776, 25.124)	0.005
Gestational stage (vs. First trimester)		24.765		
Second trimester	2.156	24.713	8.633 (3.690, 20.195)	<0.001
Third trimester	1.987	16.105	7.293 (2.764, 19.248)	<0.001
ART conception vs. Natural conception	1.278	9.298	3.588 (1.578, 8.158)	0.002
Number of antenatal visits (vs. ≤5)		7.142		
6 to 10	0.510	4.467	1.665 (1.038, 2.671)	0.035
≥11	1.004	5.921	2.728 (1.216, 6.124)	0.015
Hypothyroidism (yes vs. no)	0.658	4.956	1.931 (1.082, 3.446)	0.026

ART: Assisted reproductive technology. * Regression model with adjustments for pregnant women’s age, employment during pregnancy, household register, income, pre-pregnancy BMI, pregnancy status, number of pregnancies, number of fetuses, number of abortions, planned pregnancy, and iron deficiency anemia; the test level of the introduction of variables α = 0.05, the inspection level of excluding variables α = 0.10.

## Data Availability

The data presented in this study are available on request from the corresponding author.
